# The expressions of MIF and CXCR4 protein in tumor microenvironment are adverse prognostic factors in patients with esophageal squamous cell carcinoma

**DOI:** 10.1186/1479-5876-11-60

**Published:** 2013-03-08

**Authors:** Lin Zhang, Shu-Biao Ye, Gang Ma, Xiao-Feng Tang, Shi-Ping Chen, Jia He, Wan-Li Liu, Dan Xie, Yi-Xin Zeng, Jiang Li

**Affiliations:** 1 State Key Laboratory of Oncology in South China, Sun Yat-Sen University Cancer Center, Guangzhou, China; 2 Department of Biotherapy, Sun Yat-Sen University Cancer Center, 651 Dongfeng East Road, Guangzhou, 510060, China; 3 Department of Clinical Laboratory, Sun Yat-Sen University Cancer Center, Guangzhou, China; 4 Department of Intensive Care Unite, Sun Yat-Sen University Cancer Center, Guangzhou, China

**Keywords:** Esophageal squamous cell carcinoma, Tumor microenvironment, MIF, CXCR4, Prognosis

## Abstract

**Background:**

Tumor-derived cytokines and their receptors usually take important roles in the disease progression and prognosis of cancer patients. In this survey, we aimed to detect the expression levels of MIF and CXCR4 in different cell populations of tumor microenvironments and their association with survivals of patients with esophageal squamous cell carcinoma (ESCC).

**Methods:**

MIF and CXCR4 levels were measured by immunochemistry in tumor specimens from 136 resected ESCC. Correlation analyses and independent prognostic outcomes were determined using Pearson’s chi-square test and Cox regression analysis.

**Results:**

The expression of CXCR4 in tumor cells was positively associated with tumor status (*P* = 0.045) and clinical stage (*P* = 0.044); whereas the expression of CXCR4 in tumor-infiltrating lymphocytes (TILs) and the expression of MIF in tumor cells and in TILs were not associated with clinical parameters of ESCC patients. High MIF expression in tumor cells or in TILs or high CXCR4 expression in tumor cells was significantly related to poor survival of ESCC patients (*P* < 0.05). Multivariate analysis showed that the expression of MIF or CXCR4 in tumor cells and the expression of MIF in TILs were adverse independent factors for disease-free survival (DFS) and overall survival (OS) in the whole cohort of patients (*P* < 0.05). Furthermore, the expression of MIF and CXCR4 in tumor cells were independent factors for reduced DFS and OS in metastatic/recurrent ESCC patients (*P* < 0.05). Interestingly, the expressions of MIF and CXCR4 in tumor cells and in TILs were significantly positively correlated (*P* < 0.05), and the combined MIF and CXCR4 expression in tumor cells was an independent adverse predictive factor for DFS and OS (*P* < 0.05).

**Conclusion:**

The expressions of MIF and CXCR4 proteins in tumor cells and TILs have different clinically predictive values in ESCC.

## Background

Esophageal squamous cell carcinoma (ESCC) is one of the major histopathological subtypes of esophageal cancer. ESCC is the fourth most prevalent malignancy in China and a leading cause of cancer-related death, and its overall five-year survival rate is less than 30% [1]. It has been reported that the molecular markers related to tumor cell growth and metastasis, the function of the tumor infiltrating-lymphocytes (TILs), and the interaction between tumor cells and infiltrated immune cells in tumor microenvironments have been evaluated for their contribution to the prognoses of ESCC patients in recent studies, except the traditional prognostic factors determined at diagnosis, such as TNM stage and cell differentiation [2-7]. However, reliable markers for disease development and prognosis are still lacking in ESCC. To date, it has been revealed that the expression levels of some over-expressed genes within tumor microenvironments are related to the prognosis of ESCC, such as interleukin 17 (IL-17), SKP2, Foxp3 and Tumor necrosis factor (TNF)-related apoptosis-inducing ligand (TRAIL); however the results are still conflicting [8-13].

The macrophage migration inhibitory factor (MIF) is a 115-amino acid secreted cytokine that is involved in a number of pathological conditions, including autoimmunity, obesity and cancer [14]. The primary MIF receptor is CD74, and CD74 can bind to CD44 to form a receptor complex and mediate the transduction of MIF signaling [15]; However, CD74 can also form complexes with the C-X-C chemokine receptor type 2 (CXCR2) and type 4 (CXCR4) to transmit MIF signals to integrins in inflammatory cells [16,17]. Recent studies have demonstrated that MIF and CXCR4 were overexpressed in a number of cancers, including gastric cancer, breast cancer, prostate cancer, colon cancer and nasopharyngeal carcinoma [18-26]. However, the expression pattern of MIF and CXCR4 proteins in tumor microenvironments and their impact on the survival of cancer patients are still unclear.

Therefore, we evaluated the expression of MIF and its receptor CXCR4 protein in tumor cells and TILs of tumor microenvironment in 136 resected ESCC specimens using immunohistochmeistry staining. The correlations between the expression levels of MIF and CXCR4 in different cell subsets in tumor microenvironment and prognostic factors were assessed to determine the clinical relevance and predictive value of the MIF and CXCR4 expression in different cell subsets of tumor microenvironments of ESCC.

## Methods

### Patient selection

One hundred and thirty-six ESCC patients who underwent surgery at Sun Yat-Sen University Cancer Center in Guangzhou City of China from November of 2000 to December of 2002 were involved in this retrospective study. None of the patients had received anticancer treatment prior to surgery, and all of the patients had histologically confirmed primary ESCC. The patients had a median age of 62 years (range, 35 to 90 years); 111 (81.6%) were males and 25 (18.4%) were females. There were 74 (54.4%) cases of Stage I and II tumors and 62 (45.6%) cases of Stage III and IV tumors based on the International Union against Cancer 2002 TNM staging system and WHO classification criteria [27]. Of the 136 patients, 103 (75.7%) had died. The patients’ clinical parameters are detailed in Additional file 1: Table S1. The tumor specimens were obtained as paraffin blocks from the Pathology Department of our cancer center and clinical data were obtained from hospital records after surgery. The follow-up data from the 136 patients with ESCC in this study were available and complete. The OS was defined as the time interval from the date of surgery to the date of cancer-related death or the end of follow-up (December 2011), and the DFS was defined as the time interval from the date of surgery to the date of tumor recurrence or tumor metastasis. This study was approved by the Research Ethics Committee of the Sun Yat-Sen University Cancer Center.

### Reagents and antibodies

The following primary antibodies were used in this study: mouse anti-human MIF (ab55445; Abcam, USA), mouse anti-human CXCR4 (Clone 44716; R&D Systems, Minneapolis, MN), and horseradish peroxidase-labeled goat antibody against a mouse/rabbit IgG antibody (Envision; Dako, Glostrup, Denmark).

### Immunohistochemistry and assessment

The paraffin-embedded tissues were sectioned continuously into 4-μm-thick sections. The tissue sections were dewaxed in xylene, rehydrated and rinsed in graded ethanol solutions. The antigens were retrieved by heating the tissue sections at 100°C for 30 min in citrate (10 mmol/L, pH 6.0) or EDTA (1 mmol/L, pH 9.0) solution when necessary. The sections were then immersed in a 0.3% hydrogen peroxide solution for 30 min to block endogenous peroxidase activity, rinsed in phosphate-buffered saline (PBS) for 5 min, and incubated with the primary antibodies, including MIF, CXCR4 at 4°C overnight. A negative control was performed by replacing the primary antibody with a normal murine IgG antibody. The sections were then incubated with a horseradish peroxidase-labeled goat antibody against a mouse/rabbit secondary antibody at room temperature for 30 min. Finally, the signal was developed for visualization with 3, 3′-diaminobenzidine tetrahydrochloride (DAB), and all of the slides were counterstained with hematoxylin.

Two independent observers blinded to the clinicopathological information scored the MIF and CXCR4 expression levels in tumor cells by assessing (a) the proportion of positively stained cells :(0, <5%; 1, 6 to 25%; 2, 26 to 50%; 3, 51 to 75%; 4, >75%) and (b) the signal intensity: (0, no signal; 1, weak; 2, moderate; 3, strong). The score was the product of a × b. The levels of MIF and CXCR4 expression in lymphocytes were obtained by counting the positively and negatively stained lymphocytes in five to ten separate 400× high-power microscopic fields and calculating the mean percentage of positively stained lymphocytes among the total lymphocytes per field.

### Statistical analysis

All analyses were conducted with SPSS 16.0 (SPSS Inc., Chicago, IL, USA). The patients were divided into two subgroups (a high-level group and a low-level group) based on the median values of various immunohistochemical variables in our data. Pearson’s chi-square test and Fisher’s chi-square test were used to analyze the correlation between immunohistochemical variants in different cell subsets and the patients’ clinicopathological parameters. The MIF and CXCR4 expression levels were examined in tumor cells and in TILs in relation to the patients’ clinical prognosis using the Kaplan-Meier method and the log-rank survival analysis. Prognostic factors were assessed by univariate and multivariate analyses using the Cox proportional hazards model. The relationships among the expression levels of MIF and CXCR4 were assessed using Pearson’s correlation coefficient and linear regression analyses. A two-tailed *P*-value <0.05 was considered statistically significant in this study.

## Results

### Expression patterns of MIF and CXCR4 in ESCC and their correlations with clinicopathological and immunohistochemical variables

**Figure 1:**
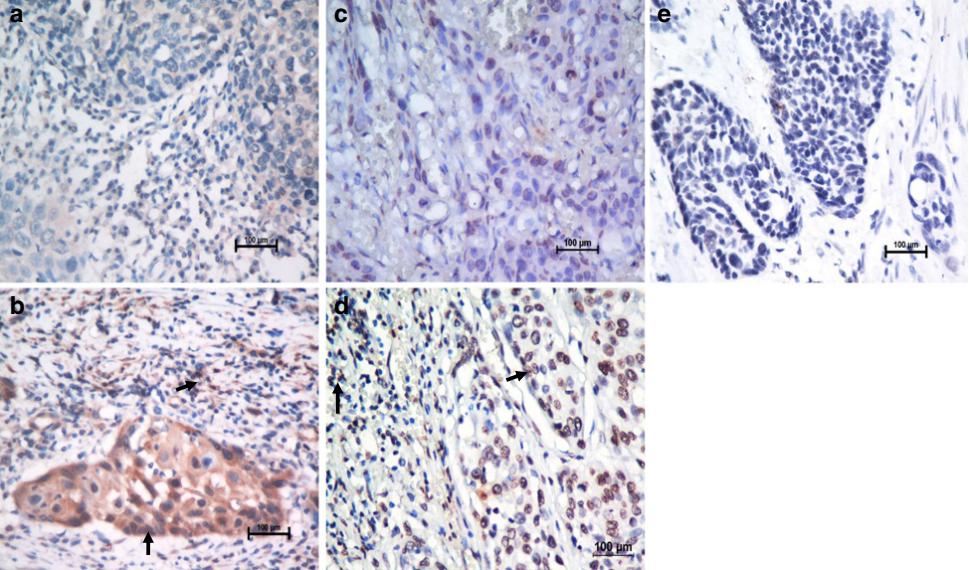
**Immunohistochemical staining for MIF and CXCR4 in human esophageal carcinoma.** Our data showed low expression levels of MIF (**a**) and CXCR4 (**c**) (X 400) and high expression levels of MIF (**b**) and CXCR4 (**d**), compared with the negative control (**e**) (X 400), in tumor tissues from patients with ESCC. The arrows point to the positive staining of tumor cells or TILs.

In the present study, the protein expression levels of MIF and CXCR4 were examined in tumor specimens from 136 patients with ESCC. MIF was expressed in the cytoplasm of tumor cells and TILs, and CXCR4 was expressed in the nucleus or cytoplasm and cell membrane of tumor cells and the cell membrane and cytoplasm of TILs (Figure 1). Based on the criteria described in the Methods section, high expression levels of MIF and CXCR4 in tumor cells were noted in samples from 73 (53.7%) and 47 (34.6%) of the 136 patients, respectively. The mean percentage and the range of the percentage of patients with TILs positive for MIF or CXCR4 expression per high-power light microscopic field were 33% (range, 0 to 92%) and 20% (range, 0 to 78%), respectively, among the 136 patients assessed (Additional file 2: Table S2).

The associations between clinicopathological features and immunohistochemical variables in different cell subsets of the tumor microenvironment in samples from 136 ESCC patients are summarized in Table 1. In the present study, the patients were divided into two groups (a high-level group and a low-level group) based on the medians of immunohistochemical variable values in diverse cell subsets. High expression levels of MIF in tumor cells were not correlated with clinicopathological variables, whereas high expression levels of CXCR4 in tumor cells were positively closely correlated with T status (*P* = 0.045) and clinical stage (*P* = 0.044). Furthermore, the expression levels of MIF and CXCR4 in TILs were not related to any of the clinicopathological parameters, including age, gender, WHO grade, T status, N status and clinical stage.

**Table 1:** Association of the expression of MIF, CXCR4 and clinicopathologic parameters in 136 patients with ESCC

**Clinicopathologic parameter**	**Case**	**Expression in tumor cells**				**Expression in TILs**			
		**High level expression of MIF (%)**	***P***^**a**^	**High level expression of CXCR4 (%)**	***P***^**a**^	**High level expression of MIF (%)**	***P***^**a**^	**High level expression of CXCR4 (%)**	***P***^**a**^
**Age**									
**≤62 (y)**	71	35 (49.3%)	0.284	26 (36.6%)	0.597	31 (43.7%)	0.122	38 (53.5%)	0.391
**>62 (y)**	65	38 (58.5%)		21 (32.3%)		37 (56.9%)		30 (46.2%)	
**Gender**									
**Female**	25	11 (44.0%)	0.283	9 (36.0%)	0.867	14 (56.0%)	0.507	17 (68.0%)	0.075^b^
**Male**	111	62 (55.9%)		38 (34.2%)		54 (48.6%)		51 (45.9%)	
**WHO grade**									
**G1**	40	27 (67.5%)	0.087	13 (32.5%)	0.669	22 (55.0%)	0.579	19 (47.5%)	0.448
**G2**	59	30 (50.8%)		19 (32.2%)		30 (50.8%)		33 (55.9%)	
**G3**	37	16 (43.2%)		15 (40.5%)		16 (43.2%)		16 (43.2%)	
**T status**									
**T1-2**	44	22 (50.0%)	0.552	10 (22.7%)	0.045*	19 (43.2%)	0.271	24 (54.5%)	0.463
**T3-4**	92	51 (55.4%)		37 (40.2%)		49 (53.3%)		44 (47.8%)	
**N status**									
**N0**	69	36 (52.2%)	0.721	20 (29.0%)	0.165	32 (46.4%)	0.391	37 (53.6%)	0.391
**N1**	67	37 (55.2%)		27 (40.3%)		36 (53.7%)		31 (46.3%)	
**Clinical stage**									
**I-II**	74	41 (55.4%)	0.659	20 (27.0%)	0.044*	37 (50.0%)	1.00	41 (55.4%)	0.168
**III-IV**	62	32 (51.6%)		27 (43.5%)		31 (50.0%)		27 (43.5%)	

Note: *P < 0.05, a, Pearson’s X^2^ test. b, Fisher’s X^2^ test.

### Immunohistochemical variables in diverse cell subsets and patient survival

**Figure 2:**
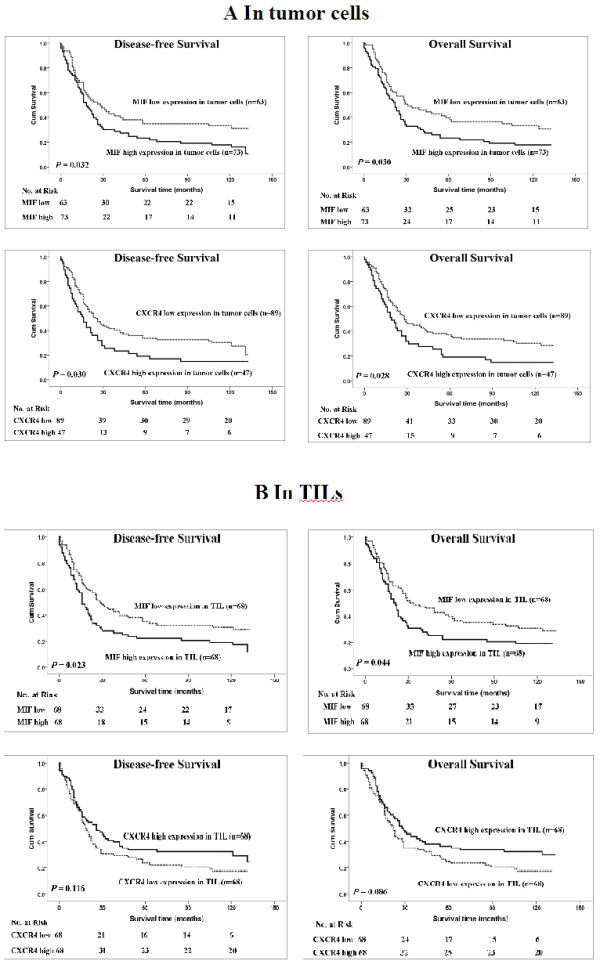
**Kaplan-Meier survival analysis in patients with ESCC.** (**A**) Disease-free survival and overall survival curves for patients according to the low and high expression levels of immunohistochemical variables in tumor cells. (**B**) Disease-free survival and overall survival curves for patients according to the low and high expression levels of immunohistochemical variables in TILs.

Among the 136 patients with ESCC, the median survival time was 25 months (range: 0 to 33 months). The cumulative five-year OS rate and DFS rate of the patients in this study were 29 and 31%, respectively (data not shown). The statistical analysis showed a significant negative correlation between DFS, OS and the expression levels of MIF in tumor cells and TILs and CXCR4 in tumor cells (*P* < 0.05, Figure 2).

The univariate analysis demonstrated that high expression levels of MIF (*P* = 0.032 and *P* = 0.030) or CXCR4 (*P* = 0.030 and *P* = 0.028) in tumor cells were noticeably correlated with reduced DFS and OS and that high expression level MIF (*P* = 0.023 and *P* = 0.044) in TILs were also significantly associated with decreased DFS and OS; however, the high expression of CXCR4 was weakly correlated with improved DFS and OS (*P* > 0.05) (Table 2). As expected, and as shown in Table 2, clinicopathological parameters such as gender, WHO grade, nodal status and TNM stage are also of prognostic value. Furthermore, we determined that, with the exception of classical prognostic factors such as gender and WHO grade, the expression of MIF or CXCR4 in tumor cells and the MIF expression in TILs were independent predictors of DFS and OS according to the multivariate Cox model analysis (Table 3).

**Table 2:** Univariate analysis of DFS and OS in 136 patients with ESCC

**Variables**	**DFS (n=136)**		**OS (n=136)**	
	**HR (95% CI)**	***P***	**HR (95% CI)**	***P***
**Age, years (**≤**62/>62)**	0.682 (0.462-1.005)	0.053	0.714 (0.483-1.054)	0.090
**Gender (male/female)**	0.452 (0.256-0.798)	0.006*	0.417 (0.232-0.747)	0.003*
**WHO Grade (1/2/3)**	1.362 (1.053-1.763)	0.019*	1.324 (1.023-1.715)	0.033*
**Tumor (T) status (1-2/3-4)**	1.569 (1.019-2.416)	0.041*	1.508 (0.976-2.332)	0.064
**Nodal (N) status (0/1)**	2.095 (1.415-3.101)	<0.001*	1.998 (1.346-2.965)	0.001*
**TNM stage (I-II/III-IV)**	1.346 (1.110-1.633)	0.003*	1.318 (1.085-1.601)	0.005*
**MIF in tumor cells (low/high)**	1.518 (1.028-2.242)	0.036*	1.532 (1.034-2.269)	0.033*
**CXCR4 in tumor cells (low/high)**	1.537 (1.035-2.283)	0.033*	1.550 (1.041-2.307)	0.031*
**MIF in lymphocytes (low/high)**	1.548 (1.053-2.275)	0.026*	1.481 (1.003-2.185)	0.048*
**CXCR4 in lymphocytes (low/high)**	0.738 (0.501-1.085)	0.122	0.715 (0.485-1.055)	0.091

Note: * *P* < 0.05.

**Table 3:** Multivariate Cox analyses for DFS and OS of 136 patients with ESCC

**Variables**	**DFS (n=136)**		**OS (n=136)**	
	**HR (95% CI)**	***p***	**HR (95% CI)**	***p***
**MIF in Tumor cells (low/high)**	1.689 (1.132-2.521)	0.010*	1.619 (1.084-2.418)	0.018*
**CXCR4 in Tumor cells (low/high)**	1.708 (1.126-2.591)	0.012*	1.612 (1.072-2.425)	0.022*
**MIF in Lymphocytes (low/high)**	1.473 (0.999-2.172)	0.050*	1.523 (1.027-2.259)	0.037*
**Combination of MIF and CXCR4 in tumor cells (low/mid/high)**	1.338 (1.064-1.683)	0.013*	1.263 (1.009-1.583)	0.042*

Note: The Cox proportional hazards regression model contained the significantly different factors in univariate analysis, including gender, WHO grade, T status, N status and TNM stage. HR, hazard ratio; CI, confidence interval; * means *P* < 0.05.

### The expression levels of MIF and CXCR4 in diverse cell subsets and the survival of patients with metastatic/recurrent ESCC

**Figure 3:**
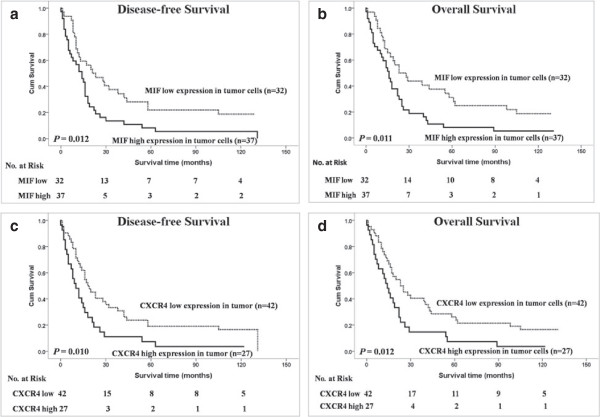
**Kaplan-Meier survival analysis in patients with metastatic/recurrent ESCC.** Disease-free survival and overall survival curves for metastatic/recurrent ESCC patients with low and high expression levels of MIF in tumor cells (**a** and **b**). Disease-free survival and overall survival curves for metastatic/recurrent ESCC patients with low and high expression levels of CXCR4 in tumor cells (**c** and **d**).

Among the 136 patients with ESCC, there were 67 (49.3%) patients with locoregional ESCC and 69 cases (50.7%) with metastatic/recurrent ESCC. The univariate analysis demonstrated that the high expression levels of MIF (*P* = 0.012 and *P* = 0.011) or CXCR4 (*P* = 0.010 and *P* = 0.012) in tumor cells were significantly correlated with poor DFS and OS in patients with metastatic/recurrent ESCC (Figure 3) but no association with locoregional ESCC (Data not shown).

### The correlation of combined expression levels of MIF and CXCR4 in diverse cell populations and survivals of patients

**Figure 4:**
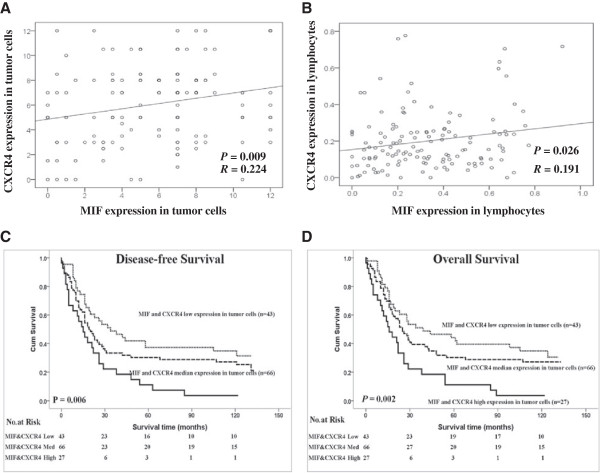
**Correlation analysis between the MIF and CXCR4 expressions in different cell populations and survival curves for ESCC patients according to their expression levels of MIF and CXCR4 in tumor cells.** (**A**) The expression levels of MIF and CXCR4 in tumor cells were significantly positively correlated (*P* = 0.009, *R* = 0.224). (**B**) The expression levels of MIF and CXCR4 in TILs were significantly positively correlated (*P* = 0.026, *R* = 0.191). (**C** and **D**) Disease-free survival and overall survival curves for patients according to the combined low expression level, single high and combined high expression level of MIF and CXCR4 in tumor cells and TILs.

In the current study, Pearson’s correlation coefficient and a linear regression analysis were applied to evaluate the correlations between the expression levels of MIF and CXCR4 in tumor cells and TILs. The MIF expression levels in tumor cells and in TILs were significantly positively correlated with the CXCR4 expression levels in tumor cells and in TILs (*P* = 0.009, *R* = 0.224 and *P* = 0.026, *R* = 0.191, respectively), as shown in Figure 4A and 4B. Furthermore, the patients with the double high expression levels of MIF and CXCR4 in tumor cells had the worst DFS and OS compared to the patients with single high expression level of MIF or CXCR4 in tumor cells or double low expression level of MIF and CXCR4 in tumor cells (*P* = 0.002 and *P* = 0.006, respectively, Figure 4C and 4D). Furthermore, the combined expression of MIF and CXCR4 in tumor cells was an independent predictive factor for DFS and OS according to the multivariate Cox model analysis (Table 2).

## Discussion

A serial of inflammatory cytokine and its receptor genes overexpress in different cell subsets of tumor microenvironments, including tumor cells and immune cells, to control the “cross talk” between tumor cells and immune cells and impact on the disease progression and clinical outcome of cancer patients [28]. MIF, a cytokine overexpressed in tumor microenvironments plays a critical role in several inflammatory conditions, as well xas in oncogenic transformation and tumor progression [29-32]. CXCR4 is the receptor of stromal cell-derived factor-1 (CXCL12/SDF-1α) and can also bind to MIF, and takes an important role in tumor progression and anti-tumor immunity. However, the association between the expression levels of MIF and CXCR4 in diverse cell populations of the tumor microenvironment and the survival of cancer patients remains ambiguous. In this context, we examined the expression pattern of MIF and CXCR4 in different cell populations in tumor tissues from 136 patients with ESCC, to determine the predictive value of the MIF and CXCR4 expressions in different cell populations within tumor microenvironment of ESCC.

High MIF levels were found in the tumors and sera of patients with different types of cancer, and MIF production has been consistently associated with the aggressiveness and metastatic potential of human tumors [33-36]. Our results suggest that MIF could be expressed in the cytoplasm of tumor cells and TILs (Figure 1). In the present study, our results demonstrated for the first time that high MIF expression in tumor cells and TILs is significantly and independently associated with poor DFS and OS in patients with ESCC (Figure 2A), as well as that high MIF expression in tumor cells is an adverse independent factor for DFS and OS in patients with metastatic/recurrent ESCC (Figure 3). Many studies have demonstrated that the biological function of MIF in tumor cells is to promote the growth of tumor cells; however, the expression of MIF in tumor tissues and patients’ clinical outcomes differed for different types of cancers [35,37-41]; our previous study showed that the increased expression of MIF in TILs within tumor microenvironments was correlated with improved outcomes for patients with nasopharyngeal carcinoma (NPC) [25]. Recent studies have indicated that MIF can induce the generation and homing of Th17 cells to the tumor microenvironments [25,42]; however, the function and clinical relevance of Th17 cells in tumor microenvironments were conflicting in different cancers [43,44]. Therefore, we think that although the expression of MIF in tumor cells is to promote the tumor cell growth as an ‘oncogenic gene’, the MIF expression in immune cells is associate with intratumoral immune response; this may explain the different impact of MIF expressions within tumor microenvironments on the survival of patients in different cancers.

CXCR4 promotes tumor progression at different levels of malignancy, including tumor growth, angiogenesis, metastatic dissemination, and homing in CXCL12-enriched cellular niches in metastatic tissues [45-47]. CXCR4 expression is a prognostic marker in various types of cancer, including acute myelogenous leukemia, breast and colon carcinomas [48,49]. Our data revealed that CXCR4 could be expressed in the nucleus, cytoplasm and cell membrane of tumor cells and TILs (Figure 1). In the current study, CXCR4 expression levels in tumor cells were positively associated with primary tumor invasion and clinical stage progression. Our results were consistent with other researchers’ findings regarding the biological functions of CXCR4 in malignant cells; namely, CXCR4 promoted malignant cell proliferation, anti-apoptosis and metastasis [45]. However, the expression of CXCR4 in tumor cells was significantly associated with poor DFS and OS in patients with ESCC or metastatic/recurrent ESCC, whereas CXCR4 expression in TILs was associated with a slightly improved DFS and OS (*P* = 0.122 and *P* = 0.091, respectively) in the ESCC patients in our study (Figure 2 and Figure 3). Our results imply that CXCR4 has different biological functions in tumor cells and lymphocytes and that high CXCR4 expression in lymphocytes can induce the homing of immune cells to tumor microenvironments to improve the number of TILs and the anti-tumor immunity of TILs in ESCC. Therefore, the combination of CXCR4 expression in both tumor cells and TILs was not associated with the survival of ESCC patients in this study (data not shown), and other studies on CXCR4 expression in tumor tissues and the clinical outcomes of ESCC patients also have reported conflicting results [50,51].

Importantly, MIF and CXCR4 expression levels in tumor cells and in TILs were positively associated (Figure 4). Our results suggest that the expression levels of MIF and CXCR4 were altered in the same way in different cell populations in the tumor microenvironments of ESCC and that the CXCR4 protein was a receptor response to MIF signaling in both immune cells and tumor cells. Interestingly, our results showed for the first time that the combined expression of MIF and CXCR4 in tumor cells was also an independent prognostic marker for ESCC patients and was strongly associated with reduced survival (Figure 4 and Table 3).

## Conclusions

The expression of tumor-derived cytokine MIF and its receptor CXCR4 were significantly associated with poor survivals of patients with ESCC; and the MIF and CXCR4 expression levels in tumor cells were independent predictive factors of survivals in patients with ESCC, as were the MIF expression level in TILs. Furthermore, the expression levels of MIF and CXCR4 in tumor cells were independent predictive factors for survivals in patients with metastatic/recurrent ESCC. Interestingly, the MIF and CXCR4 expression levels in tumor cells and in TILs were positively correlated, and the combined expression of MIF and CXCR4 in tumor cells was an adverse independent factor for survivals of ESCC patients. Therefore, the protein levels of MIF and CXCR4 in diverse cell populations within the tumor microenvironment have different clinically prognostic values in ESCC. Further studies are required to confirm our results in a large number of patients with ESCC.

## Abbreviations

ESCC: Esophageal squamous cell carcinoma; TILs: Tumor-infiltrating lymphocytes; DFS: Disease-free survival; OS: Overall survival; TNM: Tumor node-metastasis; WHO: World Health Organization; T stage: Tumor status; N status: Lymph node metastasis.

## Competing interests

The authors have declared that they have no competing interest. The sources that funded this study played no role in the study design, data collection, data analysis, decision to publish, or preparation of the manuscript.

## Authors’ contributions

Conceived and designed the experiments: JL, YXZ. Performed the experiments: LZ, XFT, SPC, JH. Analyzed the data: JL, SBY, GM. Contributed reagents/materials/analysis tools: WLL and DX. Wrote the manuscript: JL, SBY. All authors read and approved the final manuscript.

## Supplementary Material

Additional file 1: Table S1 Clinical characteristics of 136 patients with ESCC. Click here for file

Additional file 2: Table S2 Descriptive statistics of immunohistochemical variables. Click here for file
