# Supplementary material for: The expressions of MIF and CXCR4 protein in tumor microenvironment are adverse prognostic factors in patients with esophageal squamous cell carcinoma
Source: J Transl Med. 2013 Mar 8;11:60. doi: 10.1186/1479-5876-11-60 (PMC3623724; doi:10.1186/1479-5876-11-60)
Supplement: Additional file 1: Table S1 — Clinical characteristics of 136 patients with ESCC. [file 1479-5876-11-60-S1.doc]

**Supplemental file**

**Table S1 Clinical characteristics of 136 patients with ESCC**

| **Characteristics** | **No. (%)** |
| --- | --- |
| **Total cases** | 136 |
| **Age (years)** |  |
| Median | 62 |
| Range | 35-90 |
| **Gender** |  |
| Male | 111 (1.6%) |
| Female | 25 (18.4%) |
| **WHO degree** |  |
| G1 | 40 (29.4%) |
| G2 | 59 (43.4%) |
| G3 | 37 (27.2%) |
| **Tumor (T) status** |  |
| T1 | 8 (5.9%) |
| T2 | 36 (26.5%) |
| T3 | 88 (64.7%) |
| T4 | 4 (2.9%) |
| **Lymphoid node (N) status** |  |
| N0 | 69 (50.7%) |
| N1 | 67 (49.3%) |
| **Distant metastasis (M) status** |  |
| M0 | 130 (95.6%) |
| M1 | 6 (4.4%) |
| **TNM stage** |  |
| I | 6 (4.4%) |
| IIa- IIb | 68 (50.0%) |
| III | 56 (41.2%) |
| IV | 6 (4.4%) |
| **Death** |  |
| No | 33 (24.3%) |
| Yes | 103 (75.7%) |
