# Supplementary material for: The expressions of MIF and CXCR4 protein in tumor microenvironment are adverse prognostic factors in patients with esophageal squamous cell carcinoma
Source: J Transl Med. 2013 Mar 8;11:60. doi: 10.1186/1479-5876-11-60 (PMC3623724; doi:10.1186/1479-5876-11-60)
Supplement: Additional file 2: Table S2 — Descriptive statistics of immunohistochemical variables. [file 1479-5876-11-60-S2.doc]

**Supplemental file**

**Table S2 Descriptive statistics of immunohistochemical variables**

| **Variable** | **In tumor cells** | | **In TILs** | |
| --- | --- | --- | --- | --- |
|  | **Low expression level (%)** | **High expression level (%)** | **Mean percentage (%)** | **Range of percentage (%)** |
| **MIF** | 63 (46.3%) | 73 (53.7%) | 33 | 0-92 |
| **CXCR4** | 89 (65.4%) | 47 (34.6%) | 20 | 0-78 |
